# Theoretical investigation on the adsorption configuration and ^•^OH-initiated photocatalytic degradation mechanism of typical atmospheric VOCs styrene onto (TiO_2_)_n_ clusters

**DOI:** 10.1038/srep15059

**Published:** 2015-10-12

**Authors:** Honghong Wang, Yuemeng Ji, Jiangyao Chen, Guiying Li, Taicheng An

**Affiliations:** 1 State Key Laboratory of Organic Geochemistry and Guangdong Key Laboratory of Environmental Resources Utilization and Protection, Guangzhou Institute of Geochemistry, Chinese Academy of Sciences, Guangzhou 510640, China; 2 University of Chinese Academy of Sciences, Beijing 100049, China

## Abstract

In this study, the adsorption mechanism and hydroxyl radical (^•^OH)-initiated photocatalytic degradation mechanism of styrene onto different (TiO_2_)_n_ clusters were investigated using density functional theory. Styrene, a typical model atmospheric volatile organic compound (VOC), was found to be readily adsorbed onto (TiO_2_)_n_ clusters through its vinyl group with strong chemisorption. This suggests that (TiO_2_)_n_ clusters (sub 1 nm) are able to effectively adsorb and trap styrene. Adsorbed styrene is then easily attacked by ^•^OH to form a series of vinyl-OH-adducts. Conversely, phenyl-OH-adducts and H-abstraction products are very difficult to form in this system. Kinetics calculations using canonical variational transition state theory show that temperature has little effect on the rate constants during photocatalytic degradation process. The presence of TiO_2_ does not change the degradation mechanism of styrene, but can accelerate its photocatalyic degradation rate, and the rate will increase as TiO_2_ cluster size increases; as such, the TiO_2_ nano-clusters catalyst should have the photocatalytic ability to effectively degrade styrene. This theory-based study offers insights into the catalytic effect of TiO_2_ catalyst and the photocatalytic degradation mechanism of benzene series air pollutants at the molecular level.

Volatile organic compounds (VOCs) are widely used in both industrial processes and domestic activities. Anthropogenic VOCs dominate in urban areas, with a global flux of ~110 TgC per year^1^,^2^. Benzene series pollutants are important atmospheric anthropogenic VOCs, and most of them are toxic, mutagenic and carcinogenic to humans^3^,^4^,^5^. A variety of technologies have been investigated to determine how to effectively remove benzene pollutants from the atmospheric phase^6^,^7^,^8^,^9^. Heterogeneous photocatalysis has proven to be a highly effective degradation technology purifying benzene series pollutants in the air^7^,^8^,^10^,^11^,^12^. A number of laboratory studies have investigated the feasibility of this technology^10^,^13^,^14^ and kinetics^15^,^16^ to further improve the technology and enhance degradation efficiency by developing new photocatalysts for benzene abatement^17^,^18^,^19^,^20^,^21^.

To identify the photocatalyic degradation mechanism and intermediates, it is important to assess whether more or less toxic gaseous intermediates than original compounds are produced, and determine how catalysts occupy active sites to cause the deactivation. However, only a few studies have investigated the photocatalyic degradation mechanism of benzene series pollutants. For example, An *et al*. reported the photocatalytic degradation mechanism of o-toluidine and found the produced gaseous intermediates with more mutagenic activity than the parent compound^22^. However, they did not specifically identify which product caused the mutagenic toxicity in the degradation mixture. Furthermore, unfortunately, the fragmented data provided by gas chromatography–mass spectrometry (GC–MS) provide a limited view of photocatalytic degradation intermediates. As such, GC-MS testing poses significant challenges in separating mixed intermediates and successfully identifying degradation intermediates.

Given these experimental challenges, computational approaches are viable alternatives to explore the photocatalytic degradation mechanism and kinetics of VOCs. To date, little theoretical information has been reported about the photocatalytic degradation mechanism and kinetics of benzene series pollutants. However, it has been generally accepted that the photocatalytic degradation of organics is mainly mediated by various reactive oxygen species, such as ^•^OH, ^•^O_2_^−^, photo-generated holes and electrons. In particular, ^•^OH is believed to be the most important reactive species, significantly promoting the photocatalytic oxidation efficiencies of gaseous pollutants^23^,^24^.

In this study, density functional theory (DFT) was used to systematically study the ^•^OH-initiated photocatalytic degradation mechanism and kinetics of styrene onto a series of (TiO_2_)_n_ clusters. Styrene is selected as representative of benzene series pollutants, and was used as a model compound because it is a hazardous air pollutant under the Clean Air Act^25^ and is classified as a mutagenic and carcinogenic VOC by the U.S. Environmental Protection Agency^26^. With the styrene degradation, (TiO_2_)_n_ clusters were selected as models for studying nano-clusters catalysts and complex surfaces^27^,^28^,^29^,^30^ as well as to help understand photocatalytic mechanism at the molecular level, as it is generally accepted that small clusters are more reactive than their bulk counterpart^31^,^32^. Styrene adsorption patterns onto different (TiO_2_)_n_ clusters were studied to explore photocatalytic degradation mechanism. The first goal was to obtain the adsorption patterns of styrene onto a series of (TiO_2_)_n_ clusters, because the adsorption of the pollutants onto the photocatalyst surface is an important step to influence the photocatalytic degradation efficiency of gaseous pollutants. A second goal was to verify the photocatalytic mechanism and kinetics of styrene as well as the distribution profiles of the degradation product. Also, calculated results were compared with available experimental data to assess the accuracy of theoretical model.

## Results

### Adsorption mechanism of styrene onto (TiO_2_)_n_ clusters

*Properties of (TiO*_*2*_)_*n*_ *cluster surfaces*. Studies have shown that the ideal surface of TiO_2_ has a large number of Ti–O bonds^33^. (TiO_2_)_n_ clusters consist of Ti-O bonds, and therefore represent the structures and properties of a TiO_2_ nano-clusters catalyst^34^,^35^,^36^. In this work, diverse (TiO_2_)_n_ clusters (n = 1, 2, 3, 6) exhibited interesting quantum size effects; these effects were investigated to better understand the photocatalytic degradation process of styrene. Figure S1 shows the optimized structures of these clusters with theoretical data calculated by the B3LYP, MP2 and CCSD methods as well as experimental data reported by other groups^27^,^30^.

As Figure S1 shows, for the TiO_2_ clusters with C_2v_ structures, the two Ti–O bonds are calculated as 1.65 Å. For the (TiO_2_)_2_ clusters with C_2_ _h_ groups, the two terminal Ti–O bonds are 1.63 Å. These are shorter than the two internal Ti–O bonds by 0.22 Å. The Ti–Ti bond is calculated to be 2.74 Å, which is a little different from the experimental value of 2.96 Å for rutile and 2.98 Å for anatase phase TiO_2_^37^, due to the clusters are little different from the bulk phase in the non-bulk environment experienced by Ti and O in clusters. For (TiO_2_)_3_ and (TiO_2_)_6_ clusters, the internal Ti-O bonds vary from 1.73–2.08 Å, while all terminal Ti–O bonds are approximately 1.63 Å. The (TiO_2_)_3_ cluster possesses a 3-fold coordinated O-atom, one 3-fold coordinated Ti-atom and two 4-fold coordinated Ti-atoms, and the structure of (TiO_2_)_6_ cluster is characterized by six 4-fold coordinated Ti-atoms and one 4-fold coordinated O-atom with two terminal oxygen. In clusters, Ti atoms show reduced coordination, oxygen can be 1, 2 or 3 coordinated and in each cluster there is always at least one terminal oxygen bound to one Ti. The majority of oxygen atoms are 2 coordinated.

Furthermore, to test the accuracy of the results calculated by the B3LYP/6-311 g(d,p) level, the CCSD/6-311 G(d,p) and MP2/6-311(d,p) levels also measured for the comparison. The values are listed in Figure S1 along with the experimental data^27^,^30^. The MP2 method is a typical *ab* inito method, and the CCSD method is a higher electronic correlation method than aforementioned methods. From Figure S1, the B3LYP results are found to be close to the CCSD results as well as the corresponding experimental data, with the maximum error less than 2%, whereas the maximum error calculated by the MP2 method is about 5%. For example, in TiO_2_ cluster, the Ti–O distance is 1.65 Å at the B3LYP level, 1.71 Å at the MP2 level, 1.65 Å at the CCSD level, and 1.62 Å at experimental value, respectively; the O–Ti–O bond angle is 110.8°, 106.8° and 112.3° at three levels, respectively. As Figure S1 shows, the calculated geometric parameters agree well with the available experimental data as well as early reported theoretical data^27^,^30^,^37^,^38^,^39^,^40^. This indicates that the B3LYP level is a suitable method to compute (TiO_2_)_n_ clusters.

The theoretical frequencies of (TiO_2_)_n_ clusters (n = 1, 2, 3 and 6) were calculated to better understand infrared spectroscopic peaks (Figure S2). Peaks in the spectra of (TiO_2_)_n_ clusters can be classified into three regions: (i) the peaks from 1000–1060 cm^−1^ are attributed to the stretching of terminal Ti–O bonds; (ii) the peaks between 500 and 1000 cm^−1^ are attributed to the stretching of Ti–O–Ti bonds; and (iii) the peaks at approximately 530–930 cm^−1^ are observed when three-coordinated oxygen atoms are present. For the (TiO_2_)_3_ cluster, the characteristic peaks at 569, 698, and 849 cm^−1^ correspond to the vibrations of three-coordinated O-atoms; for the (TiO_2_)_6_ cluster, the corresponding peak at 863 cm^−1^ reflects the vibrations of four-coordinated O-atoms. Bulk rutile phase shows absorptions around 357, 418, 539 and 652 cm^−1^, whereas it is around 540, 660 and 740 cm^−1^ for anatase phase^27^. The characteristic peaks of (TiO_2_)_n_ clusters are not completely consistent with the rutile or anatase, suggesting that (TiO_2_)_n_ clusters are non-bulk materials, but they may be something can be exploited. So the (TiO_2_)_n_ clusters can be computed using a quantum model to understand TiO_2_nano-clusters catalyst, and that the clusters can help illustrate the adsorption patterns and the photocatalytic process of styrene as the starting points.

#### Adsorption patterns of styrene onto TiO_2_ surface

**Figure 1:**
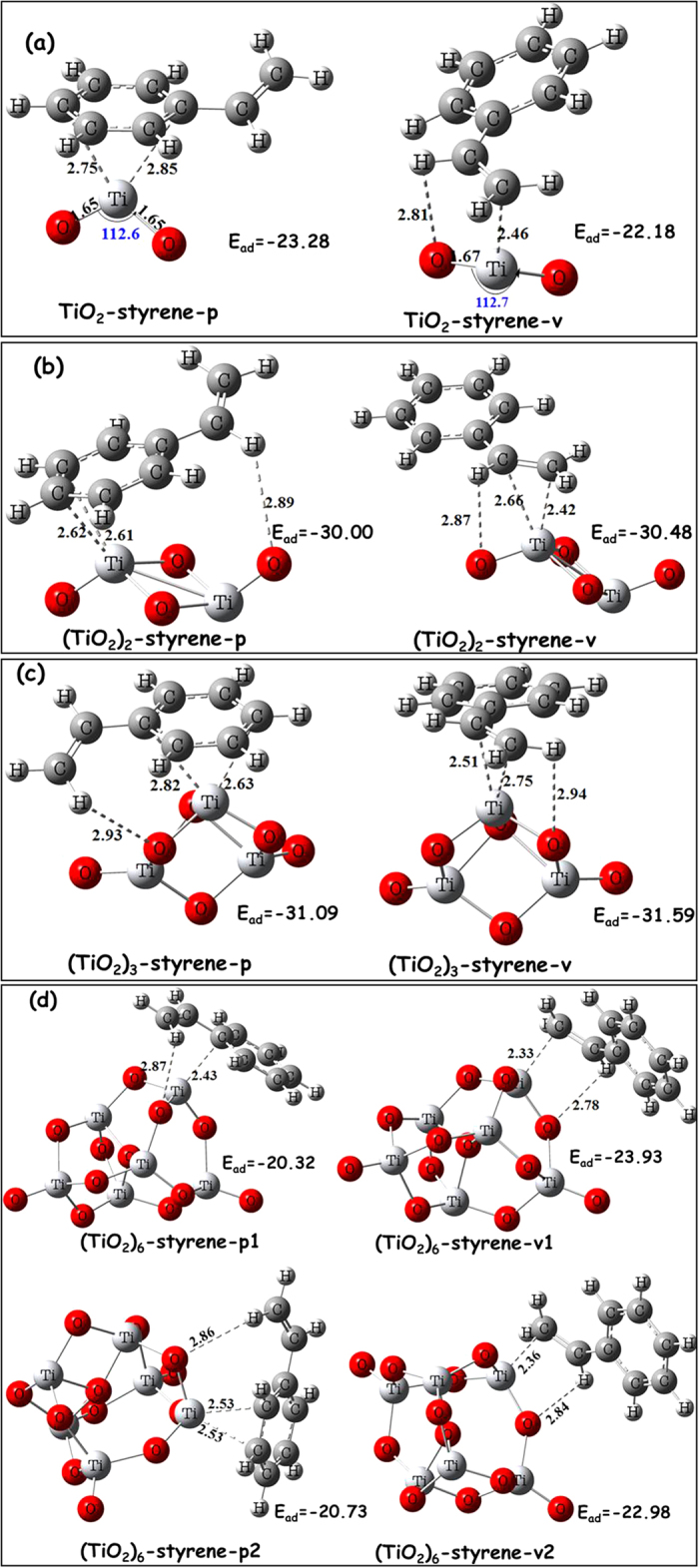
Possible adsorption configurations of styrene onto the surface of (TiO_2_)_n_ (n = 1, 2, 3, 6) clusters (in Å), with corresponding adsorption energies (E_ad_, in kcal mol^−1^).

The ability of different VOCs adsorbed onto the photocatalyst surface is critical in influencing the photocatalytic degradation efficiency of pollutants^13^. Therefore, this study explored the mechanism by which styrene is adsorbed onto a series of (TiO_2_)_n_ clusters. Figure 1 presents the optimized adsorption configurations and adsorption energies (E_ad_) of styrene adsorbed onto different (TiO_2_)_n_ clusters. For simplicity, (TiO_2_)_n_-styrene-p and (TiO_2_)_n_-styrene-v represent the adsorption patterns of the phenyl ring and vinyl group of the styrene adsorbed onto the surface of TiO_2_ clusters, respectively.

For the TiO_2_ cluster (Fig. 1a), the vinyl C_β_ and H_α_ atoms of styrene interact with Ti_cluster_ and O_cluster_ atoms of the cluster, respectively, with the distances of 2.46 and 2.81 Å to form TiO_2_-styrene-v complexes. The phenyl C_3_ and C_4_ atoms of styrene can interact with the same Ti_cluster_ atom, with Ti···C distances of 2.75 and 2.85 Å to form TiO_2_-styrene-p complexes. The E_ad_ of TiO_2_-styrene-p complex with −23.28 kcal mol^−1^ is more negative by 1.1 kcal mol^−1^ than the TiO_2_-styrene-v complex, indicating that the chemisorption of the styrene is through both the phenyl and vinyl groups of styrene. For (TiO_2_)_2_ and (TiO_2_)_3_ clusters (Fig. 1b,c), different result is obtained: the vinyl group of styrene prefers to adsorb onto these two clusters. For example, the E_ad_ is −30.00 kcal mol^−1^ for the (TiO_2_)_2_-styrene-p complex and −30.48 kcal mol^−1^ for the (TiO_2_)_2_-styrene-v complex. For the (TiO_2_)_6_ cluster (Fig. 1d), the 3-fold coordinate Ti atom can also trap styrene to form (TiO_2_)_6_-styrene-p1 and (TiO_2_)_6_-styrene-v1 complexes, while the 4-fold coordinate Ti atom can trap styrene to form (TiO_2_)_6_-styrene-p2 and (TiO_2_)_6_-styrene-v2 complexes. The Ti···C distances vary from 2.40 to 2.55 Å; while the H···O distances remain steady at approximately 2.80 Å. The E_ad_ of (TiO_2_)_6_-styrene-v complex with −23.93 kcal mol^−1^ or −22.98 kcal mol^−1^ is about 3.0 kcal mol^−1^ more negative than the (TiO_2_)_6_-styrene-p complex. Regardless of the adsorption occurring on the 3-fold coordinate Ti or 4-fold coordinate Ti of (TiO_2_)_6_ cluster, the interaction between styrene and the cluster is strongest with the (TiO_2_)_6_-styrene-v adsorption type. As discussed above, for the TiO_2_ cluster, styrene is a little readily adsorbed through the phenyl group. For the (TiO_2_)_n_ (n = 2, 3, 6) clusters, styrene prefers adsorbing onto the clusters through the vinyl group. TiO_2_ clusters with 2-fold coordinate Ti atoms are hard to find in a TiO_2_-based photocatalyst, suggesting that styrene would most likely bind with TiO_2_ nano-clusters through the vinyl group. However, as the basic structural unit of titanium oxide, the TiO_2_ cluster serves as an important quantum model to investigate TiO_2_ nano-clusters properties. In all cases, the adsorption energies are negative, varying from −20.32 to −31.59 kcal mol^−1^. Due to physorption with low adsorption energies of −1.20 to 9.56 kcal mol^−1^ and chemisorption with −9.56 to −191.30 kcal mol^−1 ^41, styrene adsorption onto the four clusters is done through spontaneous chemisorption processes. This means that the TiO_2_ photocatalyst is able to adsorb and trap styrene onto the catalysts, facilitating later degradation. The most stable adsorption structures will be focused on in the following discussion.

### _•_OH−initiated degradation of styrene onto (TiO_2_)_n_ clusters

*Degradation mechanism and kinetics*. As discussed above, although the adsorption energies of styrene onto (TiO_2_)_n_ clusters (n = 1, 2, 3, 6) differ from each other at different position, the outside phenyl and vinyl group attacked by ^•^OH are similar for four clusters. Meanwhile, increasing the cluster size significantly increases the computational time. For example, for the case of styrene adsorbed to (TiO_2_)_6_ cluster as (TiO_2_)_6_-st-p pattern, the computing time is approximately 23 d for geometry and 11 d for energy using the B3LYP/LANL2DZ method based on our workstation, respectively, while the corresponding CPU time drops to only 5 d and 16 h for the case of styrene adsorbed to TiO_2_ cluster (TiO_2_-st-p). It can be found that in the geometry calculation, the difference between CPU times of the above adsorption is not too large, but in the energy calculation, the difference is about 16 times. Considering this study, at least 15 pathways including about 500 stationary points must be calculated in OH-initiated degradation based on these adsorption models, thus the cumulative time for all of these stationary points is huge and not neglectable. As such, this study mainly focus on investigation of the ^•^OH-initiated photocatalytic degradation mechanism and kinetics of styrene onto the TiO_2_ cluster.

**Figure 2:**
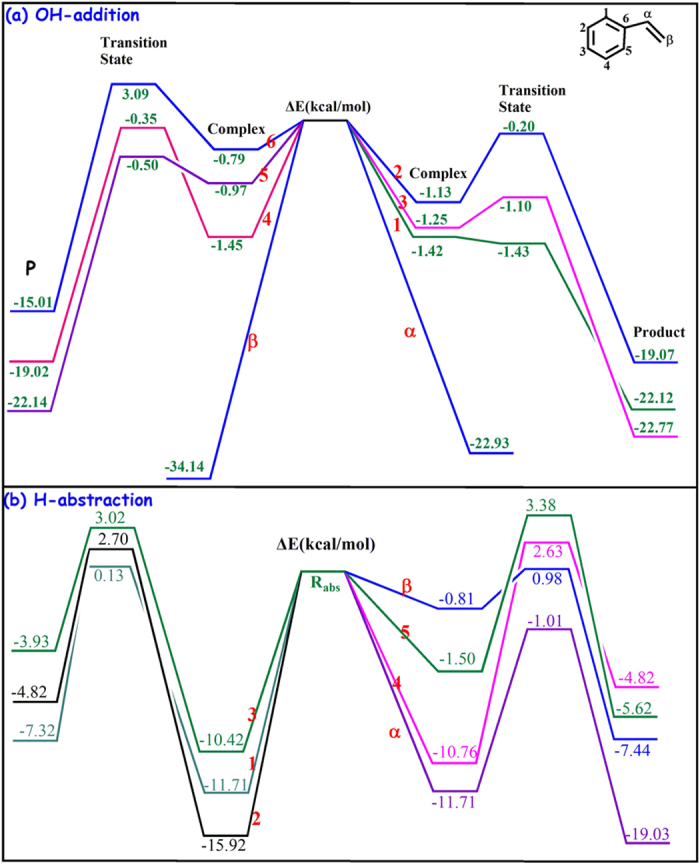
Potential Energy Surface of ^•^OH-initiated styrene degradation.

Figures 2 and S3 display the potential energy surface of the ^•^OH-initiated degradation of styrene. The figures show two kinds of channels: H-abstraction (R_abs_) and OH-addition (R_add_) channels. For the R_add_ channel (Fig. 2a), the ^•^OH attacks phenyl group and vinyl group of styrene from above. Two vinyl-OH-addition channels (R_add_α and R_add_β) are found to be barrierless processes (Figure S4), with high exothermic energies of −22.93 and −34.14 kcal mol^−1^, respectively. This means that two channels are more likely to occur on the surface of TiO_2_ cluster than the phenyl-OH-addition channels (R_add_1–6) with a barrier process. The barrier energies (ΔE) are −1.43 kcal mol^−1^ for R_add_1, −0.20 kcal mol^−1^ for R_add_2, −1.10 kcal mol^−1^ for R_add_3, −0.35 kcal mol^−1^ for R_add_4, −0.50 kcal mol^−1^ for R_add_5, and 3.09 kcal mol^−1^ for R_add_6, respectively. The ΔE of all phenyl-OH-addition channels is negative, except for the R_add_6 channels. There is a complex located at the entrance of the corresponding OH-addition channel, in which the OH is perpendicular to the benzene ring. The stabilization energy of these complexes is all about 0.79–1.45 kcal mol^−1^ stable than the corresponding reactants (Figue S2). As Fig. 2b shows, all R_abs_ channels are barrier processes and the ΔE values are positive, indicating that H-abstraction channels are difficult to occur during ^•^OH-initiated photocatalyic degradation.

Given this, the kinetics of R_add_ channel was found to be the key pathway of this study. Table 1 lists the rate constants of each R_add_ channel and the total rate constants (*k*_total_; the sum of the rate constants for each R_add_ channel) within a temperature range of 217–298 K. Table 1 shows that the total rate constant is 4.10 × 10^−10^ cm^3^ molecule^−1^ s^−1^ at 217 K, which is slightly higher than that of 3.84 × 10^−10^ cm^3^ molecule^−1^ s^−1^ at 298 K. Although the rate constant decreases as the temperature rises, this small difference suggests that temperature does not play an important role in the photocatalyic degradation process.

**Figure 3:**
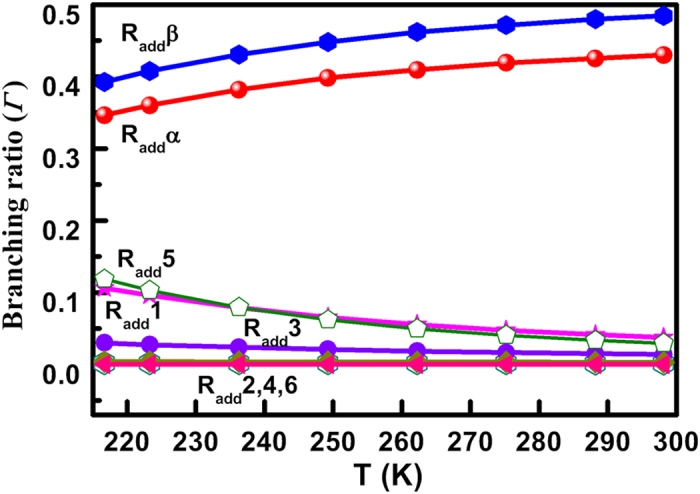
Branching ratio (*Γ*) of each ^•^OH-addition channel.

Figure 3 displays the temperature dependence branching ratio (*Γ*) for each OH-addition channel. The total *Γ* of phenyl-OH-addition channels (R_add_1–6) is less than 8% at 298 K; as the temperature decreases to 217 K, the *Γ* increases up to 30%. However, the total *Γ* of vinyl-OH-addition channels (R_add_α and R_add_β) are always more than 70% within the range of 217–298 K; even at 298 K, the Γ is 43% for R_add_α and 48% for R_add_β. Moreover, the rate constant of vinyl-OH-addition channels is calculated as 3.51 × 10^−10^ cm^3^ molecule^−1^ s^−1^ at 298 K, which is 11 times higher than that of phenyl-OH-addition channels. These results indicate that vinyl-OH-addition channels are the dominant degradation pathway, consistent with the degradation mechanism discussed above. The modified Arrhenius formulae is simulated as *k* = 4.05 × 10^−19^ T^3.13^ exp(842/T) cm^3^ molecule^−1^ s^−1^, and the activation energy is calculated as −1.67 kcal mol^−1^. This calculation aligns with the rate constant analysis that a negative effect of the temperature is found during these degradation processes. Lower activation energy also suggests that the adsorbed styrene can be easily attacked by ^•^OH and further degraded onto the TiO_2_ cluster.

#### The influence of cluster size on the photocatalytic degradation of styrene

First, the sizes of four clusters were calculated as follows: 0.344 nm for the TiO_2_ cluster, 0.455 nm for the (TiO_2_)_2_ cluster, 0.514 nm for the (TiO_2_)_3_ cluster, and 0.575 nm for the (TiO_2_)_6_ cluster. This reflects an increase in size from the TiO_2_ cluster to the (TiO_2_)_6_ cluster. To verify the role of the cluster size in the styrene degradation process, the direct correlation between the cluster diameters and the barrier energies, as well as the corresponding rate constants is calculated and shown in Table 2. R_add_β and R_add_6 channels were selected as models for the barrierless and barrier processes, respectively. As Table 2 shows, as the cluster diameters increase, the reaction energies (ΔE_p_) become more negative, leading to the progressive increase in the rate constants. For example, the reaction energy is −34.14 kcal mol^−1^ for the TiO_2_ cluster; the energy is reduced to −38.29 kcal mol^−1^ for the (TiO_2_)_6_ cluster. The corresponding rate constant increases from 1.86 × 10^−10^ to 5.13 × 10^−10^ cm^3^ molecule^−1^ s^−1^, indicating that the degradation reaction can take place more quickly. A similar result is seen with the barrier process. That is, there is a slight higher rate constant for photocatalytic degradation with an increase in (TiO_2_)_n_ clusters. However, the current study mainly focuses on the degradation of styrene onto (TiO_2_)_n_ clusters of sub 1 nm dimensions, without extending bulk or surfaces. Therefore, change in energies with the cluster size (sup 1 nm) or bulk surface need to further research to better understand the effect of TiO_2_ catalyst size on the adsorption and degradation of VOCs.

To further test the effect of cluster size on the styrene degradation mechanism, Table S1 and Figure S5 present the geometry and barrier energy parameters of each OH-addition channel for the case of the (TiO_2_)_2_ cluster. Compared with the TiO_2_ cluster, both ΔE and ΔE_p_ of each channel decrease, but the order does not change. For example, the ΔE_p_ of vinyl-OH-addition channels (R_add_α and R_add_β) channels are more negative by 7.08 and 6.08 kcal mol^−1^ than that of TiO_2_ cluster, and both of them are still the dominant channels, with a Γ of more than 90% across the assessed temperature range. Table S2 and Figure S6 show that the rate constant of vinyl-OH-addition channel is greater than that of phenyl-OH-addition channel, consistent with the TiO_2_ cluster. This suggests that an increase in the cluster size cannot change the photocatalytic degradation mechanism, although there are some differences in energy parameters. Therefore, we assume that small cluster results align with nano-clusters material results, providing insight into the photocatalytic properties of TiO_2_ catalyst at the molecular level.

#### The effect of TiO_2_ catalyst on the photocatalytic degradation of styrene

**Figure 4:**
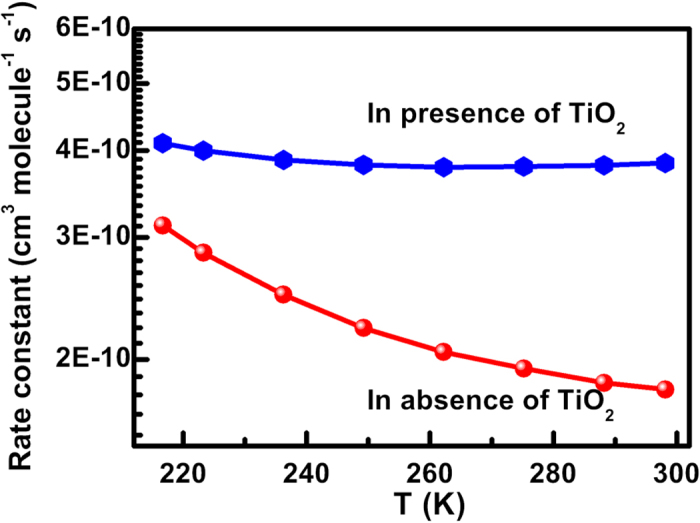
The comparison of total rate constant of ^•^OH-addition channels in the presence and absence of TiO_2_ catalyst.

To better understand the efficiency of TiO_2_ catalyst on the photocatalytic degradation of styrene, the mechanism and kinetics in the absence^42^ and presence of TiO_2_ are compared (Table S3 and Fig. 4). Table S3 shows the order of the barrier energies in the absence of TiO_2_: the ΔE(vinyl-OH-addition channel) is greater than ΔE(phenyl-OH-addition channel), which is greater than ΔE(H-abstraction channel). This order is in line with the order of that in the presence of TiO_2_, indicating that the presence of TiO_2_ does not change the mechanism. However, for the vinyl-OH-addition channel R_add_α, a change is observed between the barrier process and the barrierless process; and it’s contribution at 298 K increases from 8% in the absence of TiO_2_ to 43% in the presence of TiO_2_ (Table S3). This means that although the presence of TiO_2_ cluster can not change the mechanism, it makes the vinyl group of styrene more active than without the TiO_2_ cluster. This leads to the contribution of the vinyl-OH-addition channels to the total increase in the rate constant. For example, the total *Γ*(298 K) of two vinyl-OH-addition channels is 67% in the absence of TiO_2_ and 91% in presence of TiO_2_, respectively. As Fig. 4 shows, the rate constants in the presence of TiO_2_ are 1.3–2 times larger than those in the absence of TiO_2_ in the whole measured temperature range of 217–298 K. In summary, TiO_2_ can accelerate the degradation rate of styrene, but does not change the mechanism.

## Discussion

This study investigated the photocatalytic degradation processes of styrene onto different (TiO_2_)_n_ cluster surfaces using quantum chemistry and a theoretical dynamics model. Because the adsorption of gaseous pollutants onto the photocatalyst surfaces is a critical step to influence the photocatalytic degradation efficiency of pollutants, the adsorption mechanisms of styrene onto a series of (TiO_2_)_n_ clusters were also modeled in detail. It can be found that (TiO_2_)_n_ clusters are more favorable to trap styrene via the vinyl group with the adsorption energies of more than −20 kcal mol^−1^, and it makes the vinyl group more easily attacked by ^•^OH.

Therefore, vinyl-OH-addition channels are found to be the predominant channels for OH initiated degradation of styrene, with a more than 70% branching ratio across a temperature range of 217–298 K. The other channels, such as phenyl-OH-addition and H-abstraction channels, are small contributors; the H-abstraction channel in particular has difficulty occurring during the photocatalytic degradation process of styrene. To better understand the efficiency of TiO_2_ on the photocatalytic degradation of styrene, the mechanism and kinetics in the absence^42^ and presence of TiO_2_ clusters were compared. The results show that (TiO_2_)_n_ clusters do not change the mechanism, but can accelerate the heterogeneous photocatalytic degradation rate of styrene.

In addition, a direct correspondence between the clusters’ diameters and the barrier energies, as well as the corresponding rate constants are established to clarify the influence of the cluster size of TiO_2_ on the photocatalytic degradation rate of styrene. The results suggest that the increase of the cluster size (sub 1 nm) can enhance the degradation rate, without changing the photocatalytic degradation mechanism. These results are expected to provide an insight into the photocatalytic degradation mechanism of styrene at the molecular level, using TiO_2_ nano-clusters catalyst.

## Methods

By means of Gaussian 03 software package^43^, all the calculations for this study were carried out using density functional theory (DFT)^43^. DFT calculations can provide important information about the reaction intermediates or active species involved in the chemical reactions, and have been successfully applied to model VOCs chemistry reactions^42^,^44^,^45^. In this study, the B3LYP level is used to optimize the geometrical structures with a 6-311 G (d, p) basis set^46^,^47^ for C, H, O atoms and the standard LANL2DZ basis set^48^,^49^,^50^ for Ti atom. During the optimization, the molecule is allowed to move freely until it reaches the optimum adsorption site. Harmonic frequency analysis is conducted to identify the nature of optimized stationary points as the real local minima (without any imaginary frequency) and the transition state (with only imaginary frequency). Intrinsic reaction coordinate (IRC) theory is used to verify that the transition state structures properly connect the reactants with the products^42^,^45^,^51^. The potential energy surface (PES) is refined at the B3LYP//6-311 + G (3 df, 3pd) level to obtain energy parameters, including the barrier energy, the reaction energy, and zero point energy (ZPE). The adsorption energy (E_ad_) is calculated from the difference between the total electronic energy of surface-adsorbate complex, and the sum of the isolated molecule and the cluster surface, as shown in Eq. (1):


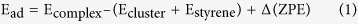


where the E_ad_ is the adsorption energy of styrene, E_complex_ is the total energy of the cluster-styrene complex, E_cluster_ is the energy of (TiO_2_)_n_ clusters, E_styrene_ is the energy of an isolated styrene molecular, and ZPE is the zero-point energy of each species. Based on this definition, a negative value corresponds to an energetically favorable adsorption process; the higher the absolute value of the adsorption energy, the more stable the adsorption configuration.

In this study, the kinetics was also calculated using generalized transition-state theory. For the barrier channels, the rate constant was obtained using Eqs. (2) and (3):


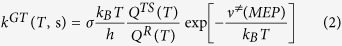



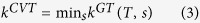


where s is the location of the generalized transition state on IRC; *k*_*b*_ and *h* are Boltzmann and Planck constants, respectively; σ is the symmetry factor related to the reaction path degeneracy; and 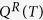 and 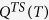 denote the total partition functions of the reactants and generalized transition state with the translational partition functions, expressed in per unit volume. The parameter 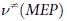 is the classical energy difference in ZPE correction, included between the transition state and the reactants along with the minimum-energy path (MEP). To include the tunneling effect, the CVT rate constant is multiplied by a multiplicative transmission coefficient computed with the so-called Winger correction^52^.

For barrierless channels, the rate constant at location *s* is along the reaction coordinate; at temperature *T*, it can be expressed as Eq. (4)


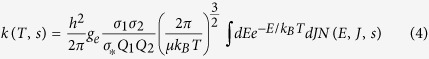


where *s* is the value of the reaction coordinate, 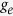 is the ratio of the electronic partition function of the transition state to the product of the electronic partition functions of reactants; *μ* is the reduced mass; *Q*_1_ and *Q*_2_ are the rotational partition functions of the reactants calculated without symmetry numbers; *J* is the unitless total angular momentum quantum number; 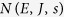 is the number of accessible states of the generalized transition state s for total energy *E* and angular momentum *Jh*; and 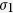, 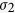,and 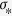 are the rotational symmetry numbers of the reactants and transition state, respectively. Rate constants of the barrierless reactions are obtained using the variable reaction coordinate variational transition state theory (VRC-VTST)^53^.

## Additional Information

**How to cite this article**: Wang, H. *et al*. Theoretical investigation on the adsorption configuration and ^•^OH-initiated photocatalytic degradation mechanism of typical atmospheric VOCs styrene onto (TiO_2_)_n_ clusters. *Sci. Rep*. **5**, 15059; doi: 10.1038/srep15059 (2015).

## Supplementary Material

Supplementary Information

## Figures and Tables

**Table 1:** Calculated rate constants of ^•^OH addition channels onto the surface of TiO_2_ within the temperature range of 217–298 K. (unit: cm^3^ molecule^−1^ s^−1^).

T (K)	k_add_1	k_add_2	k_add_3	k_add_4	k_add_5	k_add_6	k_add_α	k_add_β	k_total_
217	4.36 × 10^−11^	8.58 × 10^−13^	1.23 × 10^−11^	1.58 × 10^−12^	4.87 × 10^−11^	1.10 × 10^−15^	1.42 × 10^−10^	1.61 × 10^−10^	4.10 × 10^−10^
223	3.84 × 10^−11^	8.15 × 10^−13^	1.10 × 10^−11^	1.52 × 10^−12^	4.12 × 10^−11^	1.23 × 10^−15^	1.44 × 10^−10^	1.63 × 10^−10^	4.00 × 10^−10^
236	3.06 × 10^−11^	7.46 × 10^−13^	9.29 × 10^−12^	1.42 × 10^−12^	3.07 × 10^−11^	1.51 × 10^−15^	1.48 × 10^−10^	1.67 × 10^−10^	3.88 × 10^−10^
249	2.50 × 10^−11^	6.92 × 10^−13^	7.98 × 10^−12^	1.34 × 10^−12^	2.37 × 10^−11^	1.82 × 10^−15^	1.52 × 10^−10^	1.71 × 10^−10^	3.82 × 10^−10^
262	2.10 × 10^−11^	6.50 × 10^−13^	7.00 × 10^−12^	1.28 × 10^−12^	1.88 × 10^−11^	2.17 × 10^−15^	1.55 × 10^−10^	1.75 × 10^−10^	3.79 × 10^−10^
275	1.80 × 10^−11^	6.18 × 10^−13^	6.25 × 10^−12^	1.23 × 10^−12^	1.54 × 10^−11^	2.55 × 10^−15^	1.59 × 10^−10^	1.79 × 10^−10^	3.80 × 10^−10^
288	1.58 × 10^−11^	5.92 × 10^−13^	5.67 × 10^−12^	1.20 × 10^−12^	1.28 × 10^−11^	2.97 × 10^−15^	1.62 × 10^−10^	1.83 × 10^−10^	3.81 × 10^−10^
298	1.44 × 10^−11^	5.76 × 10^−13^	5.29 × 10^−12^	1.18 × 10^−12^	1.13 × 10^−11^	3.31 × 10^−15^	1.65 × 10^−10^	1.86 × 10^−10^	3.84 × 10^−10^

**Table 2:** Relative energies (kcal mol^−1^) and the rate constants (cm^3^ molecule^−1^ s^−1^) of OH-addition channel onto (TiO_2_)_n_ (n = 1, 2, 3, 6) clusters at 298 K.

	D(nm)	R_add_6 channel			R_add_β channel	
		ΔE	ΔE_p_	*k*_add_6	ΔE_p_	*k*_add_β
TiO_2_	0.344	3.09	−15.01	3.31 × 10^−15^	−34.14	1.86 × 10^−10^
(TiO_2_)_2_	0.455	−4.97	−17.97	7.25 × 10^−15^	−40.22	2.05 × 10^−10^
(TiO_2_)_3_	0.514	−2.67	−14.07	2.35 × 10^−15^	−38.94	5.47 × 10^−10^
(TiO_2_)_6_	0.575	−5.41	−14.55	2.25 × 10^−14^	−38.29	5.13 × 10^−10^
