# Supplementary material for: Theoretical investigation on the adsorption configuration and •OH-initiated photocatalytic degradation mechanism of typical atmospheric VOCs styrene onto (TiO2)n clusters
Source: Sci Rep. 2015 Oct 12;5:15059. doi: 10.1038/srep15059 (PMC4601031; doi:10.1038/srep15059)
Supplement: Supplementary Information [file srep15059-s1.pdf]

Supplementary Information:

Theoretical investigation on the adsorption configuration and •OH-initiated photocatalytic degradation mechanism of typical atmospheric VOCs styrene onto TiO_2 clusters

Honghong Wang[1, 2]**, Yuemeng Ji**[1]**, Jiangyao Chen**[1]**, Guiying Li**[1]**, Taicheng An**[1,*]

[1] State Key Laboratory of Organic Geochemistry and Guangdong Key Laboratory of Environmental Resources Utilization and Protection, Guangzhou Institute of Geochemistry, Chinese Academy of Sciences, Guangzhou 510640, China;

[2] University of Chinese Academy of Sciences, Beijing 100049, China.

[*] Corresponding author: **Prof. Taicheng An**

Tel: 86-20-85291501;

Fax: 86-20-85290706;

E-mail address: antc99@gig.ac.cn

The Barrierless Process for $R_{add}β$. To further confirm the barrierless of this process, the point-wise potential curve is calculated and the results is shown in Fig. S4. For the pathway $R_{add}β$, the forming C–O bond is fixed at the values from 1.3 to 2.5 Å with the interval of 0.1 Å, and the other geometric parameters were optimized for each C–O value. The minimum energy appeared at the C–O distance of 1.45 Å， which could confirm that this process is barrierless.

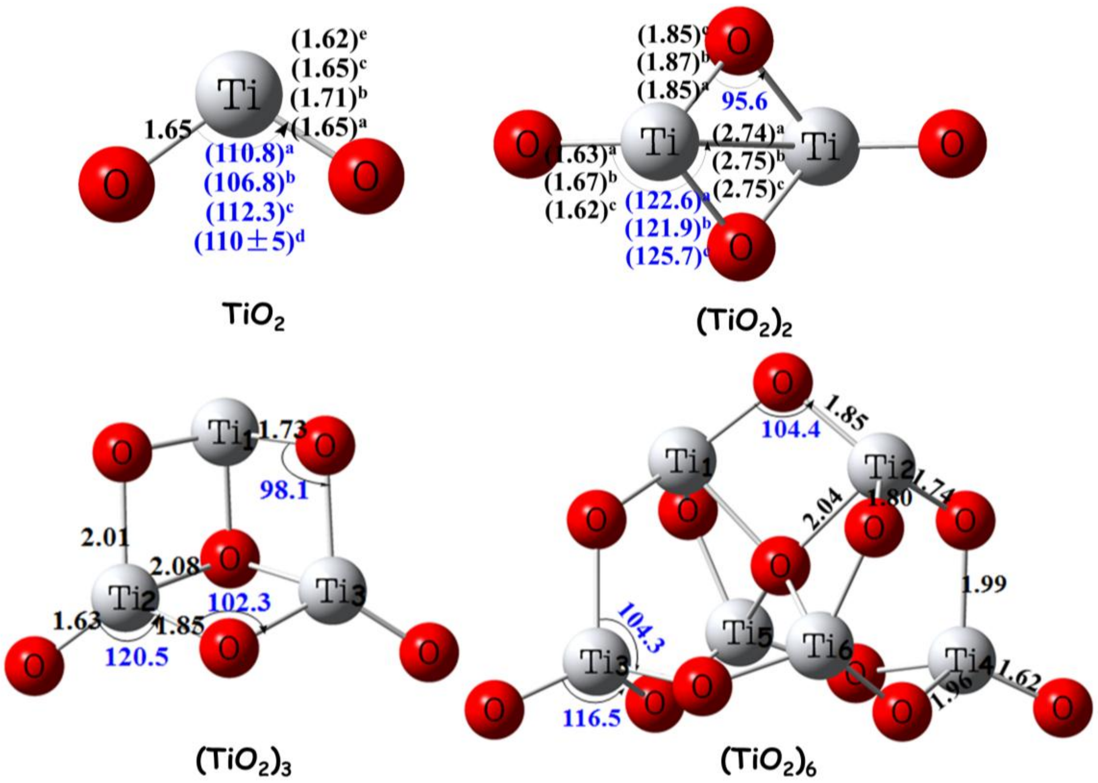

Figure S1. Optimized geometries of stable neutral $(TiO_2)_n$ clusters with n = 1, 2, 3, 6 at various levels along with the available experimental data. All bond lengths are in Å and angles in °. Calculated at a) B3LYP/6-311g(d,p), b) MP2/6-311g(d,p), c) CCSD/6-311g(d,p) d) and e) The values are experimental values from the reference of Qu et al.[1] and Calatayud et al.[2] The Ti-atoms are indicated by gray spheres and the O-atoms are indicated by red spheres.

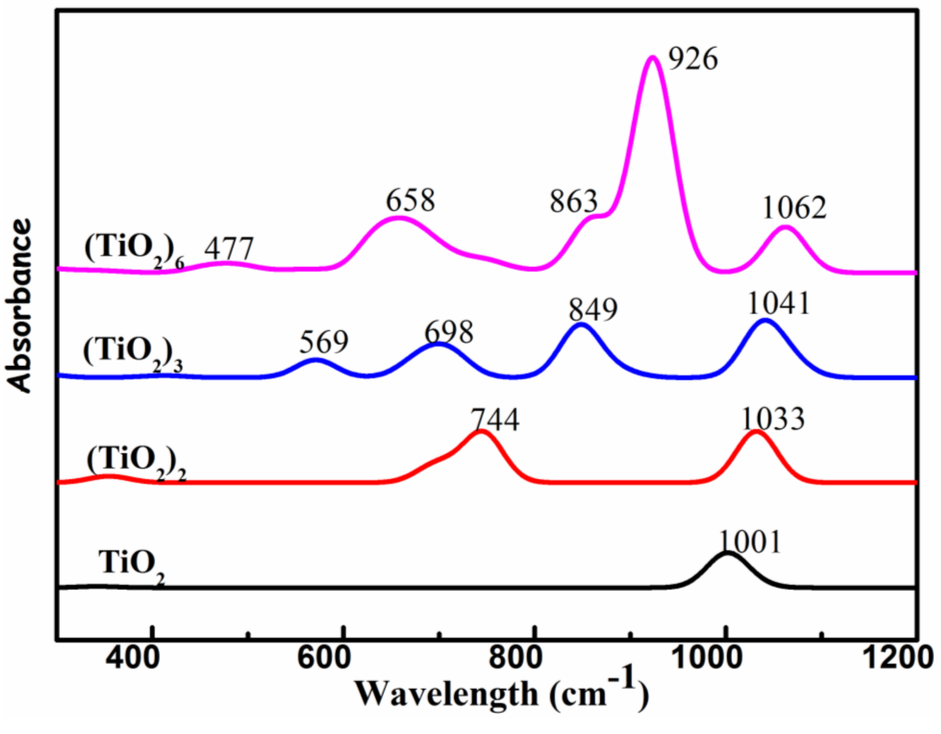

Figure S2. Infrared spectra of the most stable $(TiO_2)_n$ (n=1,2,3,6) clusters.

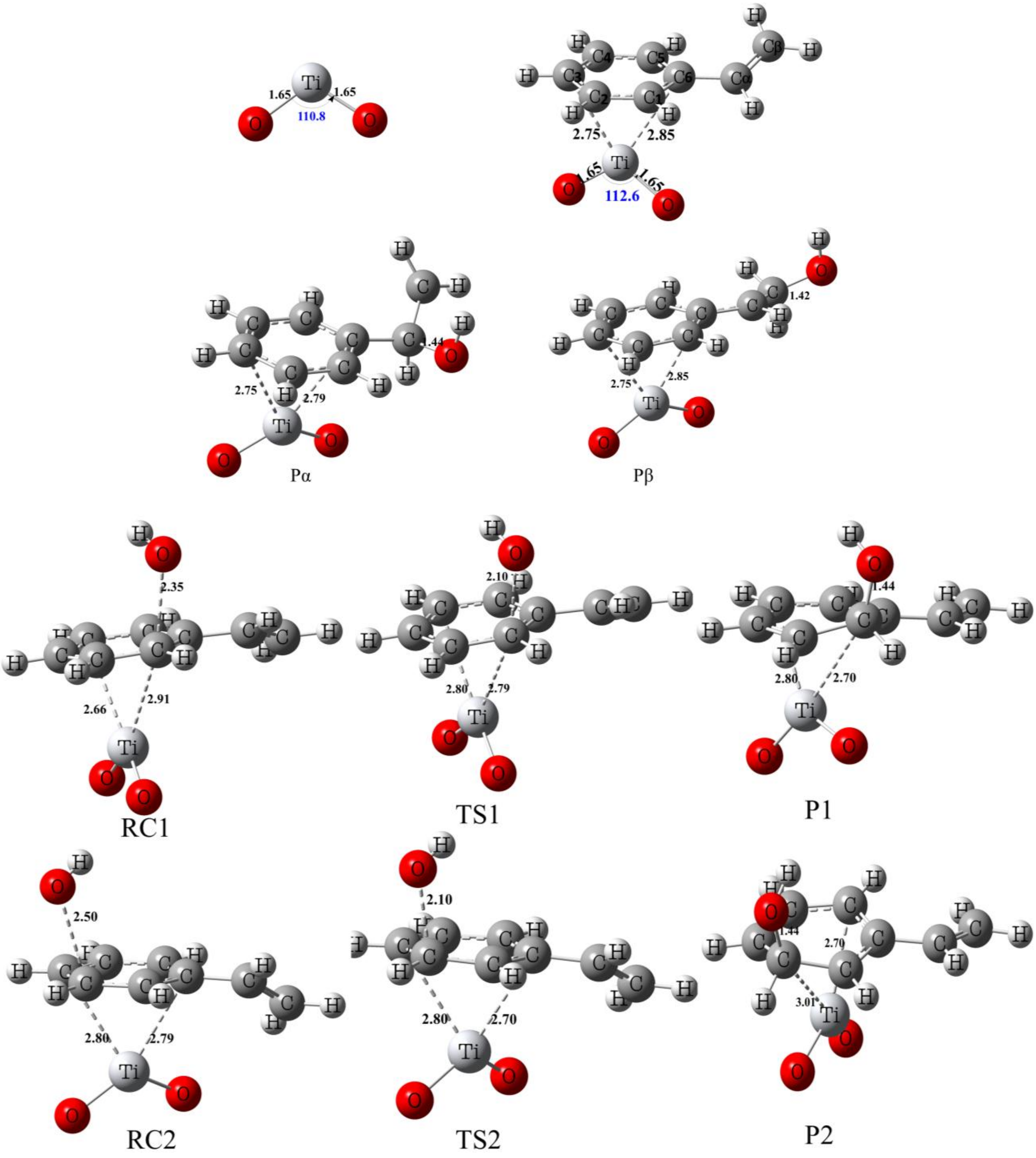

Ti
1.65
1.65
110.8
C1
C2
C3
C4
C5
C6
Cα
Cβ
2.75
2.85
112.6
Pα
1.44
2.75
2.79
Pβ
1.42
2.75
2.85
RC1
2.35
2.66
2.91
TS1
2.10
2.80
2.79
P1
1.44
2.80
2.70
RC2
2.50
2.80
2.79
TS2
2.10
2.80
2.70
P2
1.44
2.70
3.01

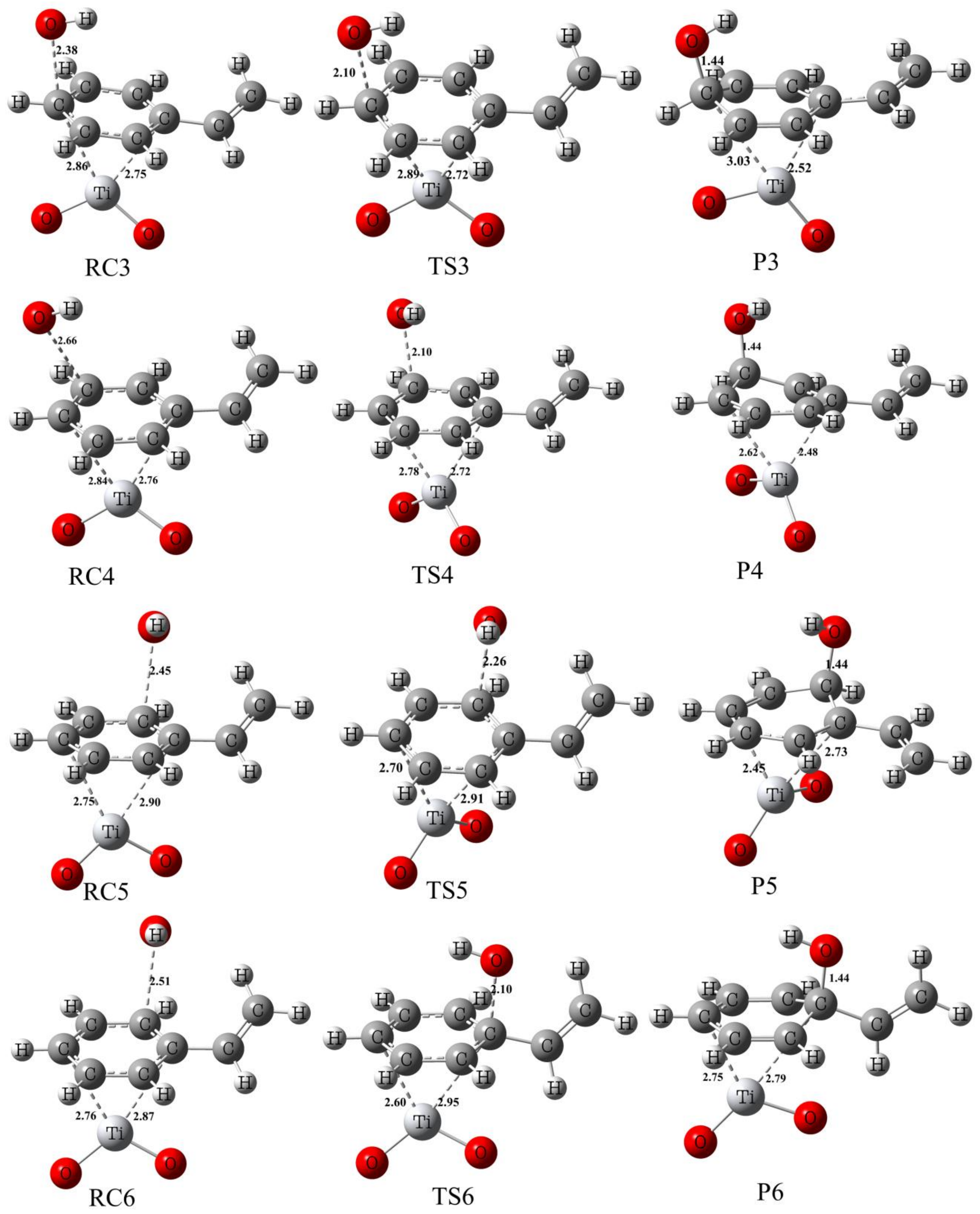

Figure S3 Optimized geometries of transition states and the products involved in the OH-addition pathways of styrene onto TiO_2 monomer surface at the B3LYP/LAN2DZ/6-311G(d,p) level. Bond lengths are in Å and angles are in °.

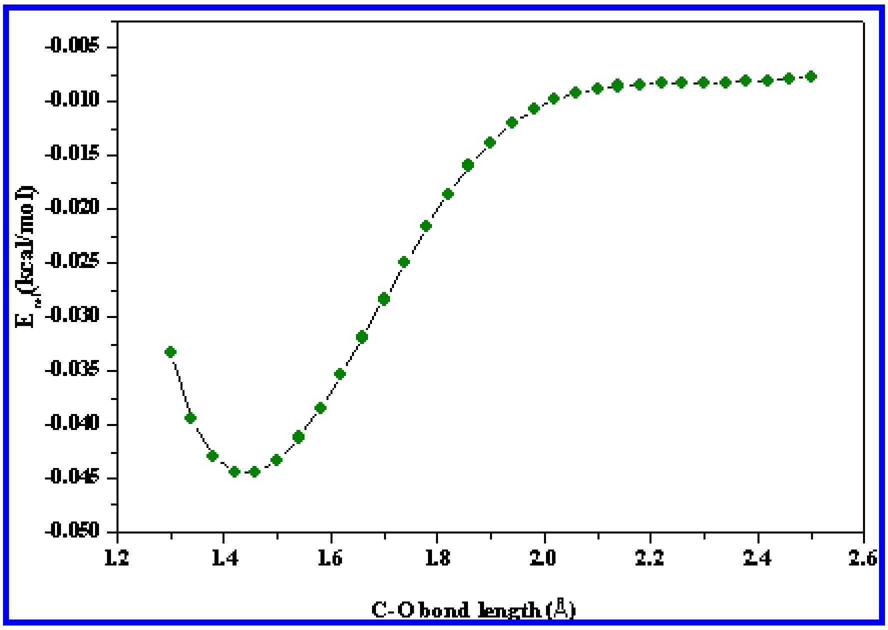

Figure S4. Potential energy curve for the formative process of C_8H_8-OH ($R_{add}\beta$) onto the surface of TiO_2 at the B3LYP/LANL2DZ/6-311G(d,p) level. The dotted line denotes the relative energy.

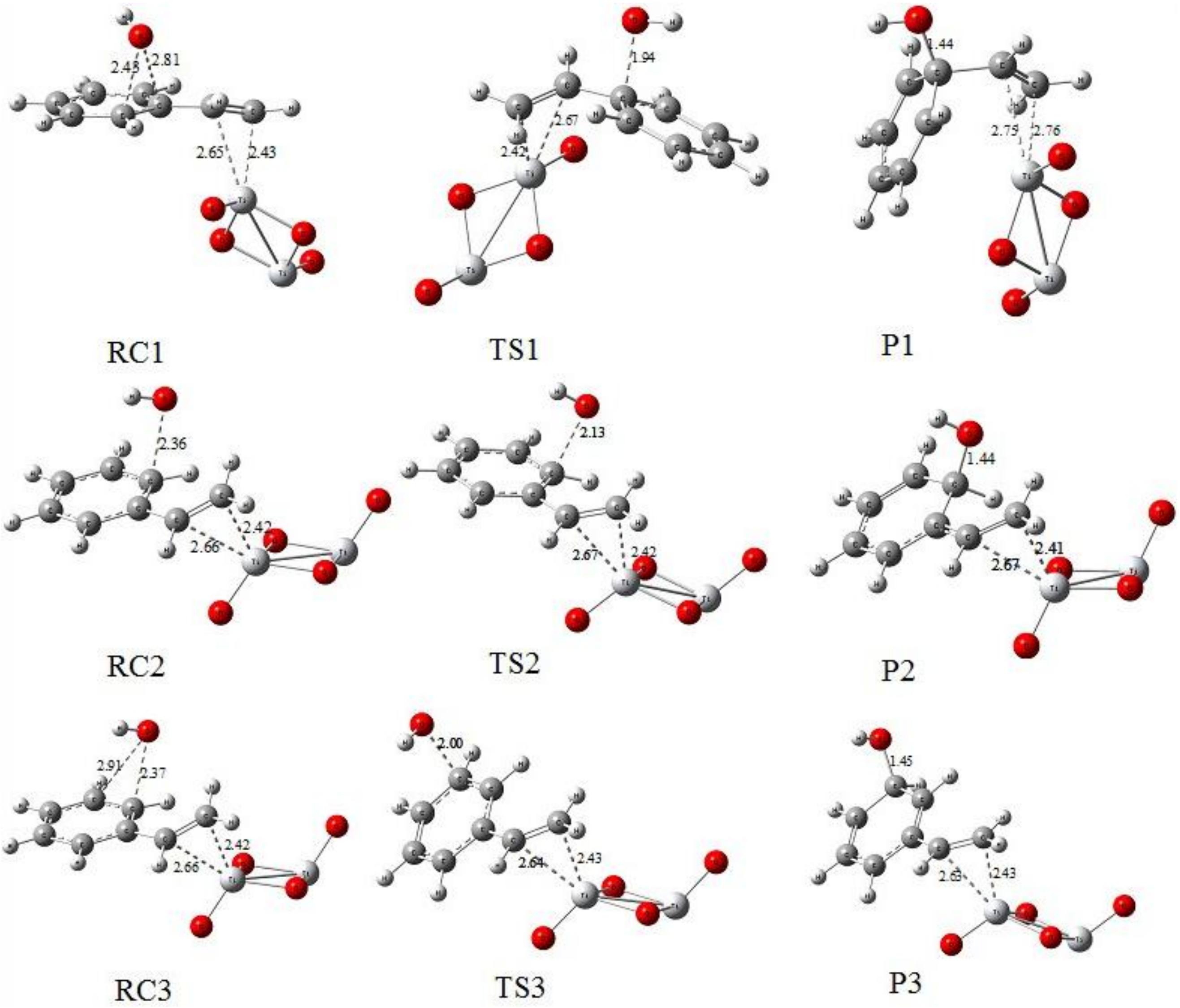
RC1
TS1
P1
RC2
TS2
P2
RC3
TS3
P3

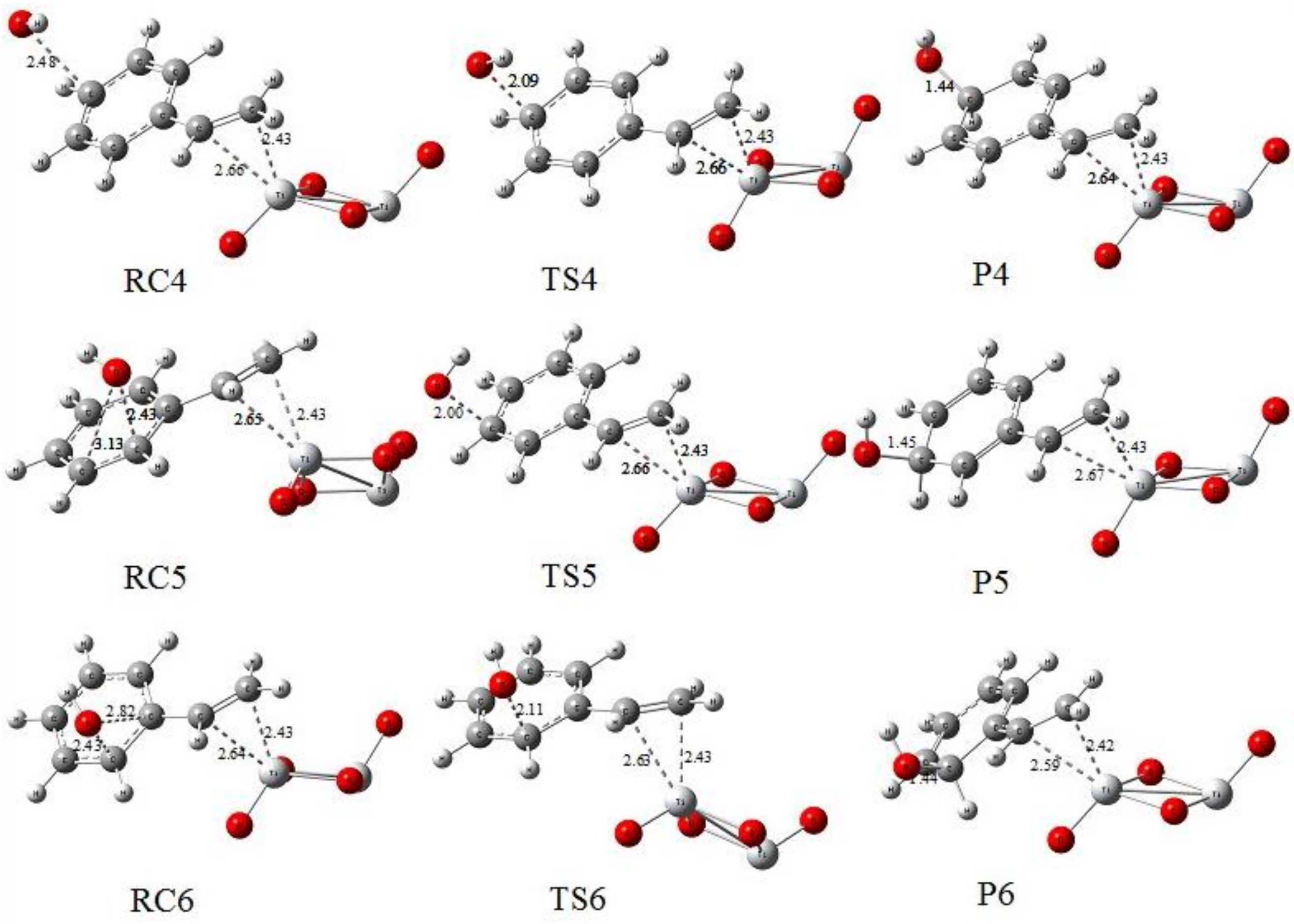

Figure S5 Optimized geometries of transition states and the products involved in the OH-addition pathways onto the surface of $(TiO_2)_2$ dimer at the B3LYP/LANL2DZ/6-311G(d,p) level. (Bond lengths are in Å and angles are in °)

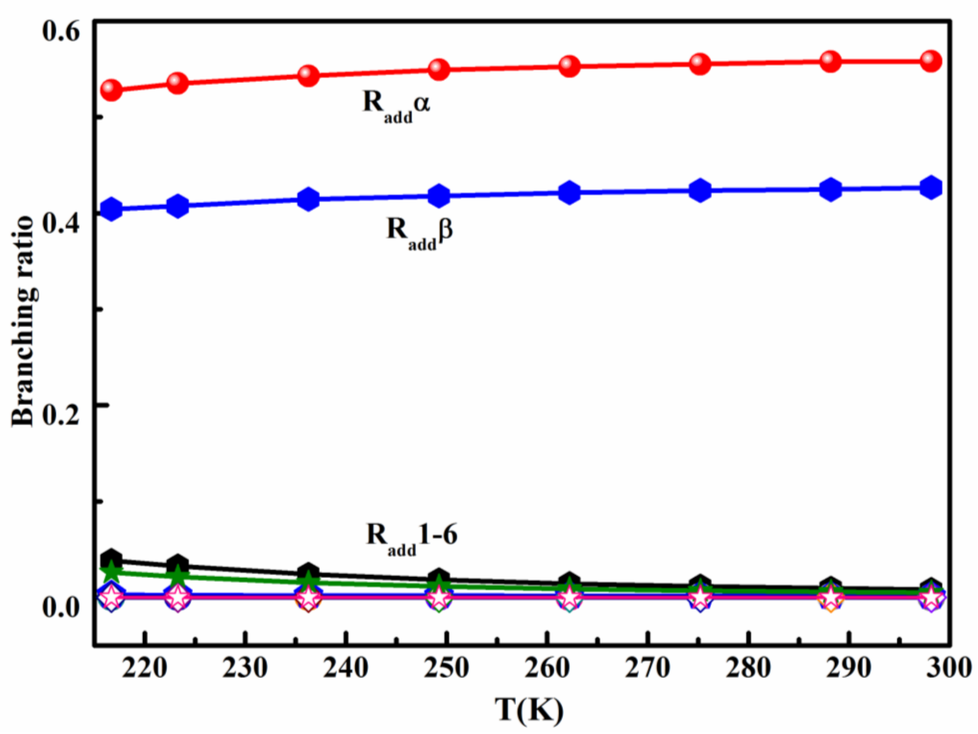

Figure S6. Branching ratios of each OH-addition pathway on the surface of $(TiO_2)_2$ cluster.

Table S1. Relative energies of OH-addition reaction on the surface of $(TiO_2)_2$ dimer. (kcal mol^{-1})

	ΔE	ΔE_p
$R_{add}1$	-8.71	-25.74
$R_{add}2$	-6.83	-20.17
$R_{add}3$	-7.70	-26.12
$R_{add}4$	-6.83	-24.98
$R_{add}5$	-8.41	-26.49
$R_{add}6$	-4.97	-17.97
$R_{add}\alpha$		-29.01
$R_{add}\beta$		-40.22

Table S2. Calculated rate constants of $^{\bullet}OH$ addition styrene onto the surface of $(TiO_2)_2$ within the temperature range of 217–298 K. (unit: cm^3 $molecule^{-1}$ s^{-1})

T (K)	$k_{add}1$	$k_{add}2$	$k_{add}3$	$k_{add}4$	$k_{add}5$	$k_{add}6$	$k_{add}\alpha$	$k_{add}\beta$	k_{total}
217	1.69×10^{-11}	1.94×10^{-13}	1.17×10^{-12}	1.10×10^{-13}	1.14×10^{-11}	3.70×10^{-15}	2.31×10^{-10}	1.77×10^{-10}	4.38×10^{-10}
223	1.44×10^{-11}	1.90×10^{-13}	1.10×10^{-12}	9.91×10^{-14}	9.45×10^{-12}	3.96×10^{-15}	2.35×10^{-10}	1.79×10^{-10}	4.39×10^{-10}
236	1.07×10^{-11}	1.86×10^{-13}	9.83×10^{-13}	9.78×10^{-14}	6.92×10^{-12}	4.47×10^{-15}	2.41×10^{-10}	1.84×10^{-10}	4.44×10^{-10}
249	8.32×10^{-12}	1.83×10^{-13}	8.94×10^{-13}	9.72×10^{-14}	5.26×10^{-12}	5.00×10^{-15}	2.47×10^{-10}	1.88×10^{-10}	4.50×10^{-10}
262	6.64×10^{-12}	1.80×10^{-13}	8.24×10^{-13}	9.70×10^{-14}	4.13×10^{-12}	5.57×10^{-15}	2.53×10^{-10}	1.93×10^{-10}	4.58×10^{-10}
275	5.43×10^{-12}	1.79×10^{-13}	7.68×10^{-13}	9.73×10^{-14}	3.33×10^{-12}	6.17×10^{-15}	2.58×10^{-10}	1.97×10^{-10}	4.65×10^{-10}
288	4.54×10^{-12}	1.79×10^{-13}	7.24×10^{-13}	9.80×10^{-14}	2.74×10^{-12}	6.79×10^{-15}	2.64×10^{-10}	2.01×10^{-10}	4.73×10^{-10}
298	4.01×10^{-12}	1.79×10^{-13}	6.94×10^{-13}	9.87×10^{-14}	2.40×10^{-12}	7.29×10^{-15}	2.68×10^{-10}	2.05×10^{-10}	4.80×10^{-10}

Table S3. Relative energies and Γ(298 K) of OH-addition reaction in presence and absence of TiO_2 catalyst. (kcal mol^{-1})

	Presence of catalyst			Absence of catalyst		
	ΔE	ΔE_p	Γ(298 K)	ΔE	ΔE_p	Γ(298 K)
$R_{add}1$	-1.43	-22.12	3.75%	-1.91	-16.49	6.57%
$R_{add}2$	-0.20	-19.07	0.15%	-0.55	-13.67	5.69%
$R_{add}3$	-1.10	-22.77	1.38%	-2.25	-19.00	9.94%
$R_{add}4$	-0.35	-19.02	0.31%	-0.35	-12.68	3.77%
$R_{add}5$	-0.50	-22.14	2.94%	-1.98	-18.04	6.35%
$R_{add}6$	3.09	-15.01	0	1.87	-9.81	0.71%
Phenyl-OH-addition			8.50%			33.03%
$R_{add}\alpha$		-22.93	43.00%	-2.66	-20.64	7.84%
$R_{add}\beta$		-34.14	48.50%		-33.60	59.10%
Vinyl-OH-addition			91.50%			66.94%

References:

1 Qu, Z. W. & Kroes, G. J. Theoretical study of the electronic structure and stability of titanium dioxide clusters (TiO_2)(n) with n=1-9. *J. Phys. Chem. B* **110**, 8998-9007, doi:10.1021/jp056607p (2006).

2 Calatayud, M., Maldonado, L. & Minot, C. Reactivity of $(TiO_2)_N$ Clusters (N=1-10): Probing Gas-Phase Acidity and Basicity Properties. *J. Phys. Chem. C* **112**, 16087-16095, doi:10.1021/jp802851q (2008).
